# Modulation of oestrone sulphate formation and hydrolysis in breast cancer cells by breast cyst fluid from British and Hungarian women

**DOI:** 10.1054/bjoc.1999.0948

**Published:** 2000-01-18

**Authors:** A Purohit, B Budai, D Y Wang, E L Willemsen, A de Winkel, D Parish, M W Ghilchik, I Szamel, M J Reed

**Affiliations:** Endocrinology and Metabolic Medicine, Imperial College School of Medicine, St Mary's Hospital, London, W2 1NY, UK; National Institute of Oncology, Ráth György Street 7–9, Budapest, 1122, Hungary; The Breast Clinic, St Mary's Hospital, London, W2 1NY, UK

**Keywords:** breast cysts, breast cyst fluid, oestrogens, oestrone sulphate, oestrone sulphatase

## Abstract

Women with gross cystic breast disease may have an increased risk of breast cancer. In this study the ability of breast cyst fluid (BCF), obtained from British or Hungarian women, to modulate oestrone sulphate (E1S) formation or hydrolysis, has been examined. For this, oestrogen receptor-positive (ER+) MCF-7 or MDA-MB-231 (ER–) breast cancer cells were employed. The formation and hydrolysis of E1S was measured using radiometric techniques. BCF from British and Hungarian women mainly inhibited E1S hydrolysis in MCF-7 cells while stimulating hydrolysis in MDA-MB-231 cells. The extent of inhibition or stimulation of E1S hydrolysis in these cells was related to the Na^+^/K^+^ratio of the BCF. There was a significant inverse relationship between the extent to which BCF samples inhibited hydrolysis in MCF-7 cells and stimulated it in MDA-MB-231 cells. BCF stimulated E1S formation in MCF-7 cells while inhibiting formation in MDA-MB-231 cells. No difference in the ability of BCF from British or Hungarian women to inhibit or stimulate E1S hydrolysis was detected in ER+ or ER– breast cancer cells. In contrast, BCF from British women stimulated E1S formation in ER+ cells (median 82%) to a significantly greater extent (*P*< 0.01) than BCF from Hungarian women (median 33%). The role that E1S has in breast cancer development remains unclear. The greater stimulation of E1S formation by BCF from British women, who have a higher risk of breast cancer than Hungarian women, suggests that it may act as a storage form of oestrogen within cells that can be activated by oestrone sulphatase. © 2000 Cancer Research Campaign


*British Journal of Cancer* (2000) **82**(2), 492–496
© 2000 Cancer Research Campaign
Article no. bjoc.1999.0948

### Modulation of oestrone sulphate formation and hydrolysis in breast cancer cells by breast cyst fluid from British and Hungarian women

**A Purohit[1], B Budai[2], DY Wang[1], EL Willemsen[1], A de Winkel[1], D Parish[1], MW Ghilchik[3], I Szamel[2] and MJ Reed[1]**

[1]Endocrinology and Metabolic Medicine, Imperial College School of Medicine, St Mary's Hospital, London W2 1NY, UK; [2]National Institute of Oncology, Ráth György Street 7–9, 1122 Budapest, Hungary; [3]The Breast Clinic, St Mary's Hospital, London W2 1NY, UK

**Summary** Women with gross cystic breast disease may have an increased risk of breast cancer. In this study the ability of breast cyst fluid (BCF), obtained from British or Hungarian women, to modulate oestrone sulphate (E1S) formation or hydrolysis, has been examined. For this, oestrogen receptor-positive (ER+) MCF-7 or MDA-MB-231 (ER–) breast cancer cells were employed. The formation and hydrolysis of E1S was measured using radiometric techniques. BCF from British and Hungarian women mainly inhibited E1S hydrolysis in MCF-7 cells while stimulating hydrolysis in MDA-MB-231 cells. The extent of inhibition or stimulation of E1S hydrolysis in these cells was related to the $Na^+/K^+$ ratio of the BCF. There was a significant inverse relationship between the extent to which BCF samples inhibited hydrolysis in MCF-7 cells and stimulated it in MDA-MB-231 cells. BCF stimulated E1S formation in MCF-7 cells while inhibiting formation in MDA-MB-231 cells. No difference in the ability of BCF from British or Hungarian women to inhibit or stimulate E1S hydrolysis was detected in ER+ or ER– breast cancer cells. In contrast, BCF from British women stimulated E1S formation in ER+ cells (median 82%) to a significantly greater extent ($P < 0.01$) than BCF from Hungarian women (median 33%). The role that E1S has in breast cancer development remains unclear. The greater stimulation of E1S formation by BCF from British women, who have a higher risk of breast cancer than Hungarian women, suggests that it may act as a storage form of oestrogen within cells that can be activated by oestrone sulphatase. © 2000 Cancer Research Campaign

**Keywords**: breast cysts; breast cyst fluid; oestrogens; oestrone sulphate; oestrone sulphatase

Breast cysts occur frequently in premenopausal women. Gross cystic breast disease (GCBD), defined as the presence of cysts more than 1 cm in diameter, is a common condition, affecting 7–10% of women in the Western world (Haagensen et al, 1981). There are two types of breast cysts. Type I have an apocrine epithelium and are filled with breast cyst fluid (BCF) that has a $Na^+/K^+$ ratio < 3, high concentration of steroid sulphates and a low albumin concentration (Miller et al, 1983). In contrast, Type II cysts are lined by a flattened epithelium and their BCF has a $Na^+/K^+$ ratio > 3, low steroid sulphate levels but a high albumin concentration (Sanchez et al, 1992; Duncan et al, 1994*a*). Women with breast cysts are generally considered to have an increased risk of breast cancer (Dixon et al, 1985, 1999; Bodian et al, 1992). Several investigations have found that there may be a higher risk associated with Type I cysts (Bruzzi et al, 1997), but this was not confirmed in a recent study (Dixon et al, 1999).

While there is evidence of an increased risk of breast cancer for women with breast cysts for Western women, it is not yet known if this association also applies to women from Eastern Europe. The incidence of breast cancer is lower in this part of the world (Armstrong and Doll, 1975). The incidence of GCBD may also be lower in Eastern Europe although there is some evidence that its incidence is increasing in Poland (Reed et al, 1994). This may possibly reflect the socio-economic changes that are taking place in that country.

*Received 2 June 1999*
*Revised 5 August 1999*
*Accepted 6 August 1999*

*Correspondence to:* MJ Reed

Breast cysts are not themselves considered to be premalignant lesions but it is possible that the fluid could influence hormone synthesis or metabolism in breast tissues adjacent to the cyst. Such a possibility has stimulated investigations to investigate the biochemical composition of BCF and also to examine its ability to modulate the actions of enzymes involved in oestrogen synthesis.

In addition to the presence of cations and steroid conjugates, BCF contains many growth factors, cytokines and proteins (Lai et al, 1990*a*; Reed et al, 1992). BCF, and some of the factors it contains, have been found to stimulate the activities of two of the key enzymes involved in oestrogen synthesis in breast cancer epithelial cells and fibroblasts. Aromatase activity, the enzyme responsible for the conversion of androstenedione to oestrone, is stimulated by BCF (Reed et al, 1993). This finding led to the identification of the cytokines, interleukin-1 (IL-1) and IL-6, as being important in regulating peripheral oestrogen synthesis. In addition, IL-6 also stimulates the conversion of oestrone to the biologically active oestrogen, oestradiol, in MCF-7 breast cancer cells (Duncan et al, 1994*b*). This reaction is mediated by oestradiol 17β-hydroxysteroid dehydrogenase Type I (E2DHI).

A third enzyme system is now considered to also have an important role in regulating blood and tissue oestrogen concentrations (Hobkirk, 1993). Much of the oestrone formed from androstenedione is converted to oestrone sulphate (E1S) by sulphotransferase enzymes. Several different sulphotransferases are present in body tissues. While there is a specific oestrogen sulphotransferase, the phenol form of phenolsulphotransferase is thought to be responsible for E1S formation in breast cancer cells (Falany et al, 1993). E1S is present at high concentration in BCF, blood and in normal and malignant breast tissues (Pasqualini et al,

1989; Orlandi et al, 1990). It can be converted back to unconjugated oestrogen by oestrone sulphatase (E1-STS). The balance between the formation of E1S and its hydrolysis will therefore have a pivotal role in regulating the availability of unconjugated oestrogens in cells and tissues. So far, however, few studies appear to have compared the effects of regulatory factors on E1S formation and hydrolysis.

In the present investigation the ability of BCF obtained from British and Hungarian women to modulate E1S formation and hydrolysis has been compared in oestrogen receptor-positive (ER+) and ER-negative (ER–) breast cancer cell lines. Such studies might reveal important clues as to the reasons for the differences in the incidence of breast cancer in British and Hungarian women. In addition, by comparing the relative effects of BCF on E1S formation and hydrolysis, further information about the role of oestrogen sulphates in breast cancer may be forthcoming.

### MATERIALS AND METHODS

### Sample collection

BCF samples were obtained by fine-needle aspiration from 17 patients (11 British and six Hungarian) attending breast clinics in London and Budapest. Their informed consent to participate in the investigations was obtained and the studies were approved by Ethics Committees at the respective hospitals. Subjects were all premenopausal and were not receiving endocrine therapy at the time of BCF collection. Samples of BCF were centrifuged at 1500 ***g*** for 10 min after collection to remove cell debris and the supernatants stored at –20°C until assayed. Hungarian BCF samples were transported to the UK on solid carbon dioxide. The ability of BCF, when stored at –20°C, to modulate sulphatase or sulphotransferase activities remained constant over a 12- to 18-month period, indicating the stability of regulatory factor(s). Sodium and potassium concentrations were measured by an indirect ion-selective electrode (Beckman Electrolyte 2 Analyser). Type I and Type II BCFs were identified by $Na^+/K^+$ ratios of < 3 or > 3.

### Cell culture

MCF-7 (ER+) and MDA-MB-231 (ER–) breast cancer cells were obtained from the American Type Culture Collection (Rockville, MD, USA). They were routinely cultured in $T_{25}$ flasks with Eagle's modified MEM with HEPES buffer (20 mM). This medium was supplemented with L-glutamine (2 mM), sodium hydrogen carbonate (10 mM), 1% non-essential amino acids, 5% (v/v) fetal calf serum (FCS) and 1% antibiotics/antimycotics. Cells were grown in this medium until approximately 40% confluent. Before adding BCF, cells were washed with phosphate-buffered saline (PBS). BCF was diluted 1:5 (v/v) with medium and sterilized by filtration. During the treatment period cells were incubated in phenol-red free medium, using the supplements as previously described with the exception that 2% (v/v) stripped FCS was used.

Triplicate flasks were treated with diluted BCF for a 48-h period after which medium was removed and intact cells were washed thoroughly with PBS before being assayed for the formation or hydrolysis of E1S. At the end of the assay period cell numbers were determined using a Coulter counter. Preliminary experiments confirmed that BCF diluted at up to 1:10 (v/v) stimulated E1-STS activity in a dose-dependent manner (Purohit et al, 1996).

### Assays for E1S formation and hydrolysis

The methods used to assay the hydrolysis (E1-STS) and formation of E1S by MCF-7 and MDA-MB-231 cells have been previously described and fully validated (Purohit and Reed, 1992; Purohit et al, 1999). Briefly, for the assay of E1-STS activity [6,7- $^3$H] E1S (2–3 nM, $7 \times 10^5$ dpm, NEN-DuPont) in serum-free medium was added to cells after which they were incubated for 20 h at 37°C. After incubation an aliquot of the medium was removed and the extent of E1S hydrolysis determined by liquid partition with toluene. [4-$^{14}$C]Oestrone (Amersham International, Aylesbury, Bucks, UK) was used to monitor procedural losses. For the assay of oestrone sulphate formation a similar procedure was employed with the exception that [6,7-$^3$H] oestrone (1–2 nM, $1 \times 10^5$ dpm, Amersham International) was used as the substrate.

Results were calculated as final product per 20 h per million cells. Mean basal E1-STS activities ($n$ = 3) in MCF-7 and MDA-MB-231 cells were 27.8 and 180 fmol per 20 h per million cells respectively. The corresponding values for E1S formation in the ER+ and ER– cells were 114 and 72.5 fmol per 20 h per million cells respectively. Because of some small variations in basal activities in cells over the duration of these investigations results have been expressed as percentage change from control cells. Representative experiments were repeated to confirm that the inter-assay variation was less than 10%. For the 48-h treatment period some small changes (± 10%) in the growth of cells was detected.

### Partial characterization of factors in BCF

BCF (Type 1) from an English subject, for which the volume exceeded 100 ml, was used to investigate the nature of the factor(s) that was able to modulate E1S formation or hydrolysis. For this, BCF (8 ml) was separated by 10 kDa cut-off membrane centrifugation (Amicon, Stonehouse, Glos., UK) and the eluent applied to a similar 3 kDa membrane, giving three fractions, i.e. > 10 kDa, 3–10 kDa and < 3 kDa. Each fraction was restored to 8 ml with cell culture medium and the effect on E1-STS activity in MCF-7 cells was assayed as described. In addition, several small chromatography columns (Superclean SPE, Sigma) were used, with BCF (1 ml) being applied to each in conditions expected to enhance binding (Solvent 1). The columns were then eluted with a solvent expected to displace bound factors (Solvent 2). The columns were cation and anion ion-exchange columns (LC-WCX and LC-SAX, Solvent 1: 0.1 M sodium phosphate buffer pH 7.4, Solvent 2: 0.1 M sodium phosphate buffer pH 7.4, 2 M sodium chloride) and phenyl and C-18 reverse phase columns (LC-pH and LC-18, Solvent 1: 0.1 M sodium phosphate buffer pH 7.4, Solvent 2: 80% acetonitrile, 0.1% trifluoroacetic acid). The bound and free fractions were taken to dryness, reconstituted in cell culture medium and their effect on E1-STS activity in MCF-7 cells assayed. Samples from columns with no BCF applied were assayed at the same time to control for solvent interference.

### Statistics

The Mann–Whitney test was used to determine the significance of differences in the ability of BCF from British or Hungarian women to modulate E1S hydrolysis or formation. Linear regression analysis was used to examine the relationships between $Na^+/K^+$ ratios and the extent of stimulation or inhibition of E1S formation

© 2000 Cancer Research Campaign 

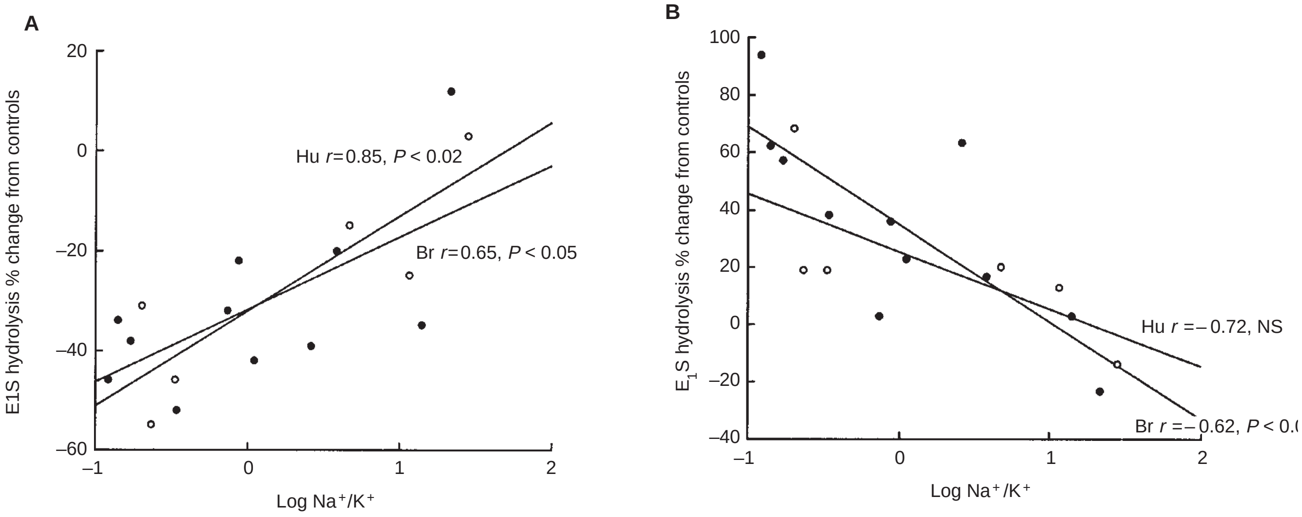

**Figure 1** Effect of breast cyst fluid from British (closed circles) or Hungarian (open circles) women on oestrone sulphatase activity in: (**A**) MCF-7 or (**B**) MDA-MB-231 breast cancer cells. Results are expressed as the percentage change compared with control cells and related to the $Na^+/K^+$ ratio of the cyst fluid

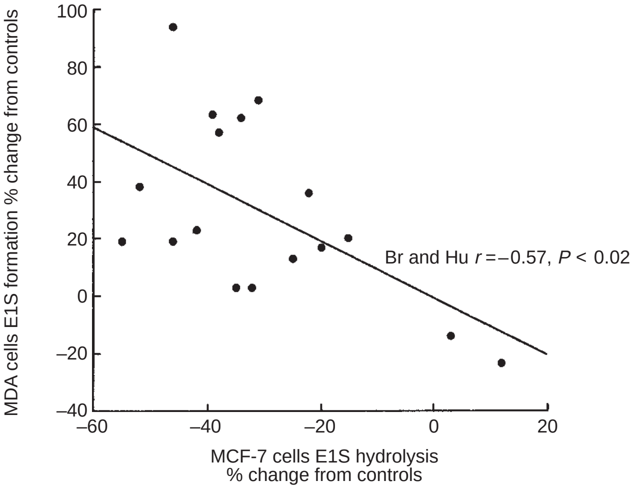

**Figure 2** Inverse correlation between the stimulation of oestrone sulphatase activity in MDA-MB-231 and inhibition in MCF-7 breast cancer cells by breast cyst fluid from British and Hungarian women or hydrolysis after logarithmic transformation of electrolyte ratios.

### RESULTS

BCFs collected from 11 British women with GCBD were initially examined for their ability of influence E1-STS activity in MCF-7 or MDA-MB-231 breast cancer cells. BCF from these women was found to modulate E1-STS activity in both cell types but in a reciprocal manner. Of the 11 samples, one stimulated E1-STS activity in the ER+ cells (12%) and inhibited activity in the ER– cells (23%). The remaining samples inhibited E1-STS activity in MCF-7 cells (18–52%) or stimulated activity in MDA-MB-231 cells (3–94%). As shown in Figure 1A and 1B, the $Na^+/K^+$ ratio was found to be related to the extent to which BCF inhibited or stimulated E1-STS activity in the ER+ and ER– breast cancer cells. That the effect did not result from the $Na^+/K^+$ concentrations in BCF was confirmed by examining the effects of different concentrations of these cations on enzyme activity. Medium in which the $Na^+/K^+$ ratios were adjusted to 0.12 or 27.0 respectively had no significant stimulatory or inhibitory effects on E1-STS (data not shown). The ability of BCF from Hungarian women to modulate E1S hydrolysis was also related to its $Na^+/K^+$ ratio (Figure 1A, 1B). Overall, there was no significant difference in the ability of BCF from British (median –35%, range –52% to 12%) or Hungarian women (median –28%, range –55% to 3%) to regulate E1S hydrolysis in ER+ cells. Similarly, the degree of stimulation in ER– cells (median values 36% and 19% respectively) did not differ significantly. The reciprocal nature of the inhibition of E1-STS in MCF-7 cells and stimulation in MDA-MB-231 cells for the British and Hungarian samples was confirmed by correlation analysis (Figure 2). There was a significant correlation ($r = –0.57$, $P < 0.02$) between the extent of inhibition or stimulation in the ER+ and ER– cells respectively.

Similar investigations were carried out to examine the effects of BCF on E1S formation in MCF-7 and MDA-MB-231 breast cancer cells. For the ER+ cells BCF from both British and Hungarian women stimulated E1S formation (Figure 3A). Stimulation of E1S formation tended to be higher by BCFs with low $Na^+/K^+$ ratios. In MDA-MB-231 cells E1S formation was inhibited by BCFs from both groups of women (Figure 3B). For these cells, however, the electrolyte ratios of the samples did not appear to influence the extent of inhibition. The extent of stimulation of E1S formation by BCF from British women in ER+ cells (median = 82%, range 46–139%) was significantly greater ($P < 0.01$) than by BCF from Hungarian women (median 33%, range 20–44%). Inhibition of E1S formation in ER– cells by BCF from British women (median –56%, range –80% to –40%) than by

 © 2000 Cancer Research Campaign

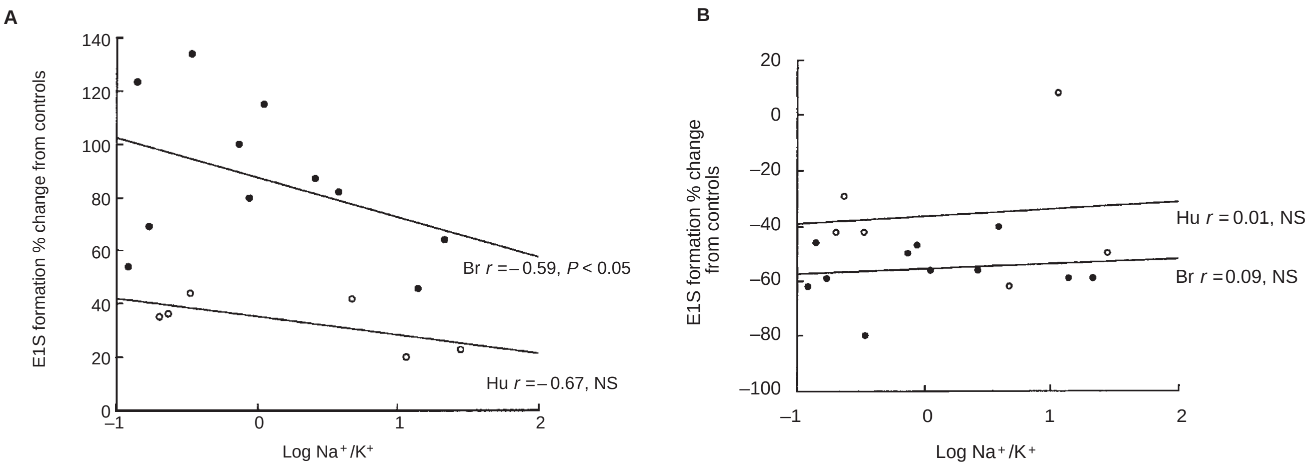

**Figure 3** Effect of breast cyst fluid from British (closed circles) or Hungarian (open circles) women on oestrone sulphate formation in: (**A**) MCF-7 or (**B**) MDA-MB-231 breast cancer cells. Results are expressed as the percentage change compared with control cells and related to the $Na^+/K^+$ ratio of the cyst fluid

BCF from Hungarian women (median –42%, range –62% to 8%) did not differ significantly.

The availability of unconjugated oestrogens to cells and tissues is likely to be governed by the balance between E1S formation and hydrolysis. The relative rates of its formation and hydrolysis in control and treated ER+/– cells were therefore examined. For untreated MCF-7 or MDA-MB-231 cells the ratios of E1S formation to hydrolysis were 4.1 and 0.4 respectively. This indicates that formation of E1S predominates in ER+ cells. Treatment of cells with BCF increased the ratio of E1S formation to hydrolysis from 4.1 to a mean value of 11.9 in MCF-7 cells. In contrast, for MDA-MB-231 cells this ratio was reduced from 0.4 to 0.14. Thus the factors present in BCF enhance E1S formation in ER+ cells while reducing it is ER– cells.

The Type 1 BCF from an English subject used in the characterization experiments was inhibitory to E1-STS activity in MCF-7 cells before fractionation. The inhibitory activity recovered after membrane centrifugation was distributed between 53% in the > 10 kDa fraction and 47% in the < 3 kDa fraction, with no activity in the 3–10 kDa fraction. The inhibitory activity bound poorly to the anion exchange column (89% of the recovered activity in the unbound fraction versus 11% in the bound and subsequently eluted fraction) but rather better to a cation exchange column (63% bound vs 36% unbound). It also bound poorly to the hydrophobic phenyl and C-18 columns (63% and 59% free respectively).

### DISCUSSION

This study has revealed that BCF from British and Hungarian women has the capacity to modulate the formation and hydrolysis of E1S in breast cancer cells. These findings confirm the results of previous preliminary investigations into the regulation of E1-STS activity by BCF (Purohit et al, 1996; Erbas et al, 1996). Overall, the abilities of BCF from British or Hungarian women to stimulate or inhibit E1S hydrolysis were similar for a given $Na^+/K^+$ ratio. In contrast, BCF from British women had a significantly greater stimulatory effect on E1S formation in ER+ cells over the range of $Na^+/K^+$ ratios examined. These results have also revealed that the extent of stimulation or inhibition is influenced by the relative concentration of $Na^+/K^+$ cations. Intriguingly, a significant reciprocal relationship was found between the ability of BCF to inhibit E1S hydrolysis in ER+ cells while stimulating it in ER– cells.

Previous investigations have found that BCF can stimulate the reduction of oestrone to oestradiol (E2DHI activity) in MCF-7 cells and aromatase activity in cultured breast fibroblasts (Lai et al, 1990*b*; Reed et al, 1993). The cytokine IL-6 was identified as a major factor in BCF that could regulate aromatase activity (Reed et al, 1992). IL-6 also stimulated E2DHI activity in MCF-7 cells (Duncan et al, 1994*b*). High concentrations of IL-6 are present in BCF with significantly higher levels present in Type II cyst fluids. IL-6 can also stimulate E1-STS in MCF-7 breast cancer cells (Purohit et al, 1999). In the present study, the BCF with the highest $Na^+/K^+$ ratio was the only sample to moderately increase, rather than inhibit, E1-STS activity in MCF-7 cells.

Steroid sulphate concentrations (e.g. dehydroepiandrosterone sulphate and E1S) can be very high in BCF and much higher than the levels of these conjugates in blood (Lai et al, 1990*b*). Steroid sulphate levels are higher in Type I BCF and they can act as competitive inhibitors of E1-STS. However, the inhibition of E1-STS activity by BCF detected in the present study is unlikely to be due to the presence of steroid sulphates. After treating cells with BCF, cells were washed with PBS before assaying for E1-STS activity. Any steroid sulphates present in the medium would be removed at this step. Inhibition of steroid sulphatase activity in epithelial cells lining cysts, by factors present in BCF, may offer a possible explanation as to why such high steroid sulphate levels are present in cyst fluid.

The most striking finding of the present study was the significantly greater stimulation of E1S formation by BCF from British women in ER+ cancer cells. The relative roles of the enzymes involved in E1S formation or hydrolysis with regard to the development of breast cancer is controversial. There is evidence that the

© 2000 Cancer Research Campaign ability to sulphate oestrogens (and therefore inactivate them) is lower in malignant than normal breast epithelium (Wild et al, 1991). Based on this observation, Anderson and Howell have postulated that sulphation is one of the means by which normal epithelial cells limit their exposure to oestrogens (Anderson and Howell, 1995). An alternative view is that the high blood and tissue concentrations of E1S acts as a storage form of oestrogens that can be made available by the action of E1-STS (Purohit et al, 1996).

In the present study the ratios of E1S formation to hydrolysis in untreated ER+ and ER– cells were 4.1 and 0.4 respectively. On treatment with BCF the ratio increased to 11.9 in MCF-7 cells while decreasing to 0.14 in MDA-MB-231 cells. It would appear, therefore, that there are fundamental differences in the way that ER+ and ER– breast cancer cells regulate E1S formation and hydrolysis. One possible explanation for these findings might be that ER+ cells need a supply of oestrogen. By stimulating E1S formation this would ensure that the cells had a stored form of oestrogen that can be reactivated by E1-STS. E1S is a polar steroid conjugate and, as such, would be unable to diffuse out of cells. In contrast, ER– MDA-MB-231 cells have no requirement for oestrogen. Hydrolysis of any oestrone formed would ensure that the unconjugated, lipid soluble steroid could rapidly leave the cell.

Some preliminary experiments were carried out in an attempt to characterize the nature of the factor(s) present in BCF that can modulate E1S formation or hydrolysis. Part of the activity had a molecular weight < 3 kDa. This would be compatible with factors such as steroids or peptides. In addition, part had an apparent molecular weight > 10 kDa which could be due either to large molecules, such as cytokines, or to binding of a smaller factor such as a steroid to high molecular weight proteins, since the separation was not carried out under denaturing conditions. As the activity did not bind to the anion ion-exchange column it is unlikely to be due to a conjugated steroid such as a sulphate. However, the partial binding to the cation exchange column suggests a possible net positive charge at pH 7.4. The relative lack of binding to the hydrophobic columns argues against the activity being an unconjugated steroid. While these are preliminary observations they are consistent with one of the inhibitory factors in BCF being a small, net positively charged peptide. Factors present in BCF with molecular weights < 3 kDa or > 30 kDa have previously been shown to stimulate E2DHI activity in MCF-7 breast cancer cells (Lai et al, 1990*b*).

In summary, the results from this investigation have revealed that BCF can modulate the formation and hydrolysis of E1S in breast cancer cells. How the ER status of cancer cells influences whether the cells predominantly form or hydrolyse E1S remains to be elucidated. As inhibitors of E1-STS may be of therapeutic value the identification of factors in BCF that inhibit the activity of this enzyme could lead to the development of novel enzyme inhibitors.

### ACKNOWLEDGEMENTS

This research was supported by a grant from the British-Hungarian Science and Technology Programme (Grant Numbers, BP/885/4/20 in UK and GB-2/95 in Hungary).

### REFERENCES

Anderson E and Howell A (1995) Oestrogen sulphotransferases in malignant and normal human breast tissue. *Endocr Rel Cancer* **2**: 227–233

Armstrong B and Doll R (1975) Environmental factors and cancer incidence and mortality in different countries with special reference to dietary practice. *Int J Cancer* **51**: 671–631

Bodian CA, Lattes R and Perzin KH (1992) The epidemiology of gross cystic breast disease of the breast confirmed by biopsy or by aspiration of cyst fluid. *Cancer Detect Prev* **16**: 95–103

Bruzzi P, Dogliotti L, Naldoni C, Bucchi L, Costantini M, Cicognani A, Torta M, Buzzi GF and Angeli A (1997) Cohort study of association of risk of breast cancer with cyst type in women with gross cystic breast disease. *Br Med J* **314**: 925–928

Dixon JM, Lumsden AB and Miller WR (1985) The relationship of cyst type to risk factors for breast cancer and the subsequent development of breast cancer in patients with breast cystic disease. *Eur J Cancer Clin Oncol* **21**: 1045–1050

Dixon JM, McDonald C, Elton RA and Miller WR (1997) Breast cancer risk with cyst type in cystic disease of the breast. *Br Med J* **315**: 545–546

Dixon JM, McDonald C, Elton RA and Miller WR (1999) Risk of breast cancer in women with palpable breast cysts: a prospective study. *Lancet* **353**: 1742–1745

Duncan LJ, Robinson GV, Ghilchik MW and Reed MJ (1994*a*) The effect of gross cystic disease fluid proteins on oestrogen synthesis in breast cancer cells. *Endocr Rel Cancer* **2**: 27–35

Duncan LJ, Coldham NG and Reed MJ (1994*b*) The interaction of cytokines in regulating oestradiol 17β-hydroxysteroid dehydrogenase activity in MCF-7 cells. *J Steroid Biochem Mol Biol* **49**: 63–68

Erbas H, Lennard TWJ and Lai LC (1996) Effect of breast cyst fluid on oestrone sulphatase activity in MCF-7 breast cancer cells. *Anticancer Res* **16**: 833–836

Falany JL, Lawing L and Falany CN (1993) Identification and characterization of cytosolic sulfotransferase activities in MCF-7 human breast cancer cells. *J Steroid Biochem Mol Biol* **46**: 481–487

Haagensen CD, Bodian C and Haagensen DE (1981) *Breast Carcinoma, Risk and Detection*. WB Saunders: Philadelphia

Hobkirk R (1993) Steroid sulfation, current concepts. *Trends Endocrinol Metab* **4**: 69–74

Lai LC, Dunkley SA, Reed MJ, Ghilchik MW, Shaikh NA and James VHT (1990*a*) Epidermal growth factor and oestradiol in human breast cyst fluid. *Euro J Cancer* **26**: 481–484

Lai LC, Coldham NG, Islam S, Reed MJ, Ghilchik MW and James VHT (1990*b*) Effect of breast cyst fluid on oestrogen 17-oxidoreductase activity in MCF-7 breast cancer cells. *Cancer Lett* **55**: 165–169

Miller WR, Dixon JM, Scott WN and Forrest APM (1983) Classification of human cysts according to electrolyte and androgen conjugate composition. *Clin Oncol* **9**: 227–232

Orlandi F, Caraci P, Puligheddu B, Torta M, Dogliotti L and Angeli A (1990) Estrone-3-sulfate in human breast cyst fluid. *Ann NY Acad Sci* **586**: 464–466

Pasqualini JR, Gelly C, Nguyen B-L and Vella C (1989) Importance of oestrogen sulphates in breast cancer. *J Steroid Biochem Mol Biol* **34**: 155–163

Purohit A, De Giovanni CV and Reed MJ (1999) The regulation of oestrone sulphate formation in breast cancer cells. *J Steroid Biochem Mol Biol* **68**: 129–135

Purohit A and Reed MJ (1992) Oestrogen sulphatase activity in hormone-dependent and hormone-independent breast cancer cells: modulation by steroidal and non-steroidal therapeutic agents. *Int J Cancer* **50**: 901–905

Purohit A, Reed MJ, Morris NC, Williams GJ and Potter BVL (1996) Regulation and inhibition of steroid sulfatase activity in breast cancer. *Ann NY Acad Sci* **784**: 40–49

Reed MJ, Coldham NG, Patel SR, Ghilchik MW and James VHT (1992) Interleukin-1 and interleukin-6 in breast cyst fluid: their role in regulating aromatase activity in breast cancer cells. *J Endocrinol* **132**: R5–R8

Reed MJ, Topping L, Coldham NG, Purohit A, Ghilchik MW and James VHT (1993) Control of aromatase activity in breast cancer cells: the role of cytokines and growth factors. *J Steroid Biochem Mol Biol* **44**: 589–596

Reed MJ, Angeli A, Thijssen JH, Milewicz A, Szamel I, Hubert J, Harlozinska-Szmyrka A, Toth J and Kornafel J (1994) Gross cystic breast disease: overview and direction for future research. *Endocr Rel Cancer* **2**: 9–13

Sanchez LM, Vizoso F, Diez-Itza I and Lopez-Otin C (1992) Identification of the major protein components in breast secretions from women with benign and malignant breast disease. *Cancer Res* **52**: 95–100

Wild MJ, Rudland PS and Back DJ (1991) Metabolism of the oral contraceptive steroids ethynyloestradiol and norgestimate by normal (Huma 7) and malignant (MCF-7 and ZR-75-1) human breast cells in culture. *J Steroid Biochem Mol Biol* **39**: 535–543

 © 2000 Cancer Research Campaign
